# Autopsy Report with Clinical and Pathophysiologic Discussion of Autosomal Dominant Adult Polycystic Kidney Disease

**DOI:** 10.1155/2014/727580

**Published:** 2014-09-17

**Authors:** Anup Hazra, Richard Siderits, Cheryl Rimmer, Noah Rolleri

**Affiliations:** ^1^Department of Pathology and Laboratory Medicine, Robert Wood Johnson Medical School, Piscataway, NJ 08854, USA; ^2^Robert Wood Johnson University Hospital-Hamilton, 1 Hamilton Health Place, Hamilton, NJ 08690, USA

## Abstract

The average weight of a kidney is approximately 135 gm, measuring on average 10 × 6 × 4 cm. In hereditary conditions, autosomal dominant and autosomal recessive polycystic kidney disease, the shape, size, and the weight can be significantly abnormal, causing progressive renal failure, often necessitating dialysis or renal transplant for survival. We report a case of adult polycystic kidney disease in a 50-year-old female without a family history, who died of complications of the disease which included accelerated hypertension, and renal and cardiac failure.

## 1. Introduction

Autosomal dominant polycystic kidney disease (ADPKD) is the more common variant (80%) of polycystic kidney disease with a prevalence of 1 to 2 cases per 400 to 1000 live births [1]. It is the most common genetic cause of chronic kidney disease and accounts for five percent of patients who initiate dialysis in the US each year [2, 3]. However more than 50% of those carrying inherited mutations in the genes responsible for ADPKD will go undetected throughout a patient's lifetime. This suggests a benign clinical course [4]. Frequency of patients presenting with ADPKD without a family history (new mutation) is about 10%.

Most cases are believed to be a result of a mutation in gene products of either the PKD1 (chromosome 16) or PKD2 (chromosome 4), which are responsible for producing the proteins polycystin-1 and polycystin-2, respectively. Mutations in PKD1 are more common (86%) and clinically more severe; the disease presents itself earlier and patients reach end stage renal disease (mean age 54 versus 74) and die at a younger age than those with mutations in PKD2 [5].

The PKD1 gene is a large and complex gene that produces a protein over 4000 amino acids in length which functions in a manner not yet completely understood [6]. Its product, polycystin-1, is an integral membrane protein found in the plasma membrane and cilia in all sites of cyst formation (kidney, liver, and pancreas) and is overexpressed in most of the cysts in ADPKD [7]. PKD2's gene product, polycystin-2 protein product, is 1000 amino acids in length and involved in cell calcium signaling. Mutations in these genes are thought to cause abnormal cell differentiation leading to various types of structural and biochemical derangements in the epithelia of renal tubules and bile and pancreatic ducts.

## 2. Report of a Case

A 50-year-old female with a long standing history of polycystic kidney disease, uncontrolled accelerated hypertension, chronic renal failure, and deep vein thrombosis (DVT) of leg was brought to the emergency room with advanced life support after being found in cardiac and respiratory arrest at home. Resuscitation efforts were unsuccessful and an autopsy was performed.

## 3. Pathologic Findings

**Figure 1:**
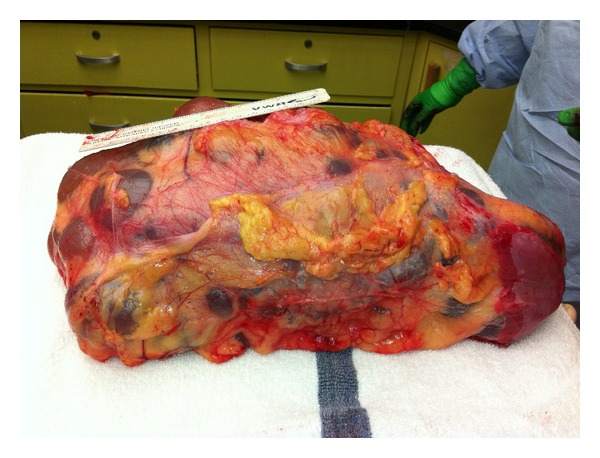
Macroscopic image of right kidney, 2400 grams.

**Figure 2:**
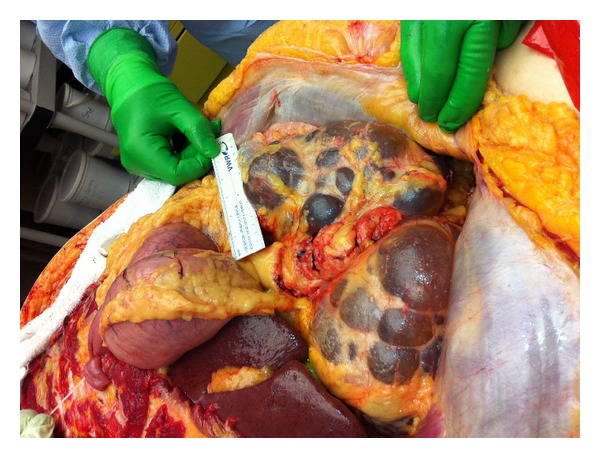
Macroscopic image of right kidney “in situ” showing bosselated cystic spaces.

**Figure 3:**
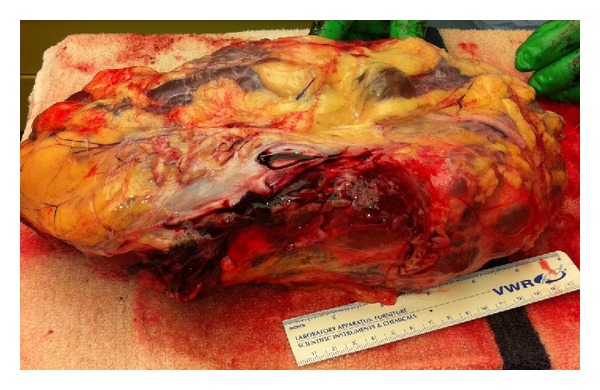
Left kidney 3450 grams.

**Figure 4:**
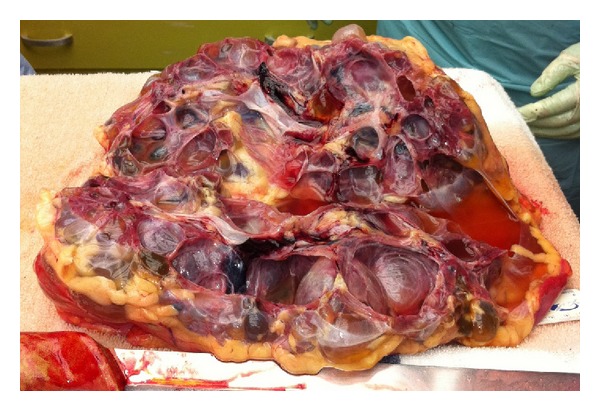
Left kidney, hemisectioned, showing smooth luminal surfaces.

Gross pathologic findings were significant for extensive bilateral polycystic kidney disease (right 2400 gm, left 3450 gm, (Figures 1, 2, 3, and 4)), hepatomegaly with small to medium cysts (2300 gm), splenomegaly (330 gm), and myocardial hypertrophy (600 gm).

Kidneys upon microscopic examination showed innumerable small to large cysts containing clear yellowish fluid with pigment laded macrophages, flattened cuboidal lining epithelium, thin cyst wall, marked chronic inflammation, frequent lymphoid aggregates, calcific deposits in tubules, and rare residual glomeruli. Multiple similar small cysts were also noted in the liver.

## 4. Comment

Cysts are believed to be formed by abnormal cell differentiation leading to excessive proliferation and fluid secretion. In the early stages of cyst formation, mutated polycystin creates abnormalities in the extracellular matrix of renal tubules that lead to dilatations that fill with glomerular filtrate. The tubular epithelial cells, immature and extensively proliferated due to dysfunctional polycystin, express abnormal amounts of electrolyte transporters that in later stages are what is responsible for fluid accumulation and cyst growth. These events lead to renal interstitial infiltration with monocytes, macrophages, and fibroblasts, subsequent fibrosis, and loss of renal function [8].

Understanding the pathogenesis behind the disease can of course help us design ways to prevent disease progression. Currently most of the treatment of ADPKD is focused around the sequelae of the disease involving strict blood pressure control and statins to reduce cardiovascular mortality. However recently there have been many preclinical and clinical trials in mechanism based therapeutics that look promising as autocrine/paracrine factors influencing cyst formation become better established [9].

Mammalian target of rapamycin (mTOR) is a serine/threonine kinase that regulates cell growth and cell cycle progression; that is, inhibition in mice models with ADPKD has been shown to preserve renal function and inhibit fibrosis [10]. In humans, mTOR inhibitors have been shown to inhibit epithelial cell proliferation in mouse models of ADPKD and may minimize the increase in kidney volumes in patients but have limited impact on slowing down the decrease in GFR [11].

Somatostatin has been shown in clinical trials to slow renal and hepatic cyst progression and halt total liver and kidney volume increase by reducing cyst fluid accumulation. It is similar to mTOR inhibitors as it has still not been shown to halt GFR decrease any greater than placebo [12].

Intracellular cyclic AMP (cAMP) plays a significant role in cystogenesis by acting as an intracellular second messenger promoting cell proliferation through MAP kinase activity and fluid secretion via the activation of chloride transporters [13]. Decreasing cAMP has been accomplished using vasopressor V2 receptor antagonists. They have been shown to inhibit cystogenesis and prevent renal enlargement and dysfunction in three different animal models with Phase II trials currently underway [10].

TGF-*β*, a protein ligand secreted by renal epithelial cells and is important for cell proliferation and differentiation, had been reported to inhibit cystogenesis in 3D cell culture. It is theorized that this is accomplished by a mechanism other than altering MAP kinase activity. The TGF-*β*2 subtype also modulates the expression of genes involved in cell-cell and cell-matrix interactions and extracellular matrix proteins that play a role in cystogenesis [14]. Studies involving its application in vivo have not yet been performed but it is an interesting target for use in preventing the progression of ADPKD.
